# Addiction, Identity, Morality

**DOI:** 10.1080/23294515.2019.1590480

**Published:** 2019-04-23

**Authors:** Brian D. Earp, Joshua August Skorburg, Jim A. C. Everett, Julian Savulescu

**Affiliations:** a Departments of Philosophy and Psychology, Yale University, New Haven, Connecticut, United States;; b Yale-Hastings Program in Ethics and Health Policy, The Hastings Center, Garrison, New York, United States;; c Uehiro Centre for Practical Ethics, University of Oxford, Oxford, United Kingdom;; d Department of Philosophy, Duke University, Durham, North Carolina, United States

**Keywords:** addiction, personal identity, true self, essential moral self, Phineas Gage effect

## Abstract

**Background:** Recent literature on addiction and judgments about the characteristics of agents has focused on the implications of adopting a “brain disease” versus “moral weakness” model of addiction. Typically, such judgments have to do with what capacities an agent has (e.g., the ability to abstain from substance use). Much less work, however, has been conducted on the relationship between addiction and judgments about an agent’s identity, including whether or to what extent an individual is seen as the same person after becoming addicted. **Methods:** We conducted a series of vignette-based experiments (total *N* = 3,620) to assess lay attitudes concerning addiction and identity persistence, systematically manipulating key characteristics of agents and their drug of addiction. **Conclusions:** In Study 1, we found that U.S. participants judged an agent who became addicted to drugs as being closer to “a completely different person” than “completely the same person” as the agent who existed prior to the addiction. In Studies 2–6, we investigated the intuitive basis for this result, finding that lay judgments of altered identity as a consequence of drug use and addiction are driven primarily by perceived negative changes in the moral character of drug users, who are seen as having deviated from their good true selves.

## Introduction

According to the U.S. Centers for Disease Control and Prevention (CDC), between 1999 and 2016, more than 630,000 people died from drug overdoses in the United States, with more than 350,000 of those due to opioids (Seth et al. 2018). Frequently described as an “epidemic,” the problem of opioid addiction has worsened in recent years, with prescription opioids drawing particular attention. In addition to calls for greater awareness of the social, economic, and public health consequences of opioid overuse (Council of Economic Advisors 2017; Smith 2018), there is also a need to understand the effects of such use at a more personal level, including its impact on families and close relationships (Egan 2018). Here, we explore an important but understudied aspect of this impact: the effects of drug abuse and addiction on judgments of personal identity.

A common refrain is that addiction changes a person. In a memoir about her son’s drug addiction, one author writes: “Six years have passed since I discovered that my son was using drugs. I [was] devastated, not to mention how worried I was about his wellbeing. *My son was not the same person anymore*” (Urzia 2014, emphasis added). Similarly, on an addiction resource webpage, one testimonial states, “My husband was a normal, kind, genuine person for the first few years of our marriage. He suddenly became addicted to cocaine and has become a different person” (Anonymous 2015). As Tobia (2017) notes, such stories are heartbreakingly common: “Many,” he writes, “have witnessed someone they loved change so profoundly that the person remaining seems an entirely different one.”

Why might people have such feelings about a loved one who has become addicted? Why do they see them as a different person (and in what sense)? One possibility seems obvious: We recognize people, in large part, by their characteristic actions, and people often act very differently when they become addicted. Imagine someone named Jim. Before abusing drugs, he was fun, outgoing, and dependable. But after the addiction took hold, he became withdrawn, irritable, and unreliable. Seemingly, these changes in demeanor could explain why Jim’s friends and family come to see him as a different person than he used to be.

However, it may not be so simple. Recent work in psychology and experimental philosophy (see Knobe 2016; Knobe and Nichols 2008; Knobe et al. 2012; Cova et al. 2018) suggests that intuitions about changed identity are shaped not just by the fact of some change in demeanor after a transformative event (cf. Paul 2014), but rather by the particular kind of change that occurs. Specifically, if a person undergoes a change in their moral attributes, they tend to be seen as far more changed as a person than if they differ in terms of almost any other identity-relevant trait: basic cognition, memory, personality, desires and preferences, and even sense perception (Strohminger and Nichols 2014).

The direction of change matters as well: When holding the magnitude of change constant, a person’s becoming morally worse, compared to morally better, makes them much less likely to be seen as the same person as they were before (Tobia 2015, 2016). One explanation for this asymmetry comes from good-true-self theory (Strohminger, Knobe, and Newman 2017). According to this theory, people typically regard others’ true selves as being fundamentally good, holding all else equal (Newman, Bloom, and Knobe 2014; De Freitas et al. 2018; Newman, De Freitas, and Knobe 2015; Bench et al. 2015). Thus, if a person undergoes a change in character from morally bad to good, people tend to interpret this not as becoming an entirely different person, but rather, finally realizing or becoming one’s true self (Bench et al. 2015; Tobia 2017). However, if one undergoes a change in the opposite direction, even if it is the same magnitude of change, one is seen as moving farther away from one’s true self, and thus as a different person (Tobia 2015).

In this work, we explore whether these recent advances in understanding lay perceptions of identity change apply to the case of addiction. This is an important line of inquiry for two reasons, one theoretical, and one more practical. The theoretical reason is that the literature on such perceptions has so far relied most prominently on fantastical or unlikely cases, such as brain-transplant thought experiments (Strohminger and Nichols 2014; but for more realistic scenarios see Strohminger and Nichols 2015) or peculiar accidents, such as the historical Phineas Gage story, wherein an unfortunate railroad worker had an iron rod shot through his head (Tobia 2015). While such extreme scenarios may be helpful for clarifying people’s intuitions about what is most central to judgments of identity persistence—that is, the extent to which an individual is regarded as essentially the same person over time—it is not yet clear whether more common, real-life cases, such as becoming addicted to drugs, fit the same pattern of intuitions. And the practical reason it is important is that if addiction does affect judgments of identity in the way we suggest, this could have profound consequences for how people understand and relate to persons with addiction in everyday life.

Here is our plan for what follows. First, we briefly discuss the literature on addiction and identity in its qualitative sense: the sense concerned with what an agent is like, or what characteristics she has. Then, we ask whether addiction might affect people’s judgments about identity persistence: the extent to which an individual is seen as the same person over time, despite changes in such personal characteristics. In this context, we expect that changes in moral characteristics will prove especially important, as will the direction of change, from morally good to bad or vice versa. We then give an overview of the empirical studies we conducted to test this idea, summarizing our main findings along the way. Near the end of the article, we situate these results in the context of wider debates about the nature of personal identity and draw some speculative conclusions about the implications of treating addiction for close relationships.

## Addiction and identity

Thinking about addiction in terms of identity is not new (Bailey 2005). Primarily, the literature in this area has been concerned with identity in a qualitative sense—what a person is fundamentally like—often cast in terms of the characteristics an individual has, or is taken to have, in virtue of her addiction (Reith 2004). Are persons with addiction free moral agents, for example, who are responsible for their behavior while under the influence of drugs, or for becoming addicted to drugs in the first place (Yaffe 2001)? Or are they passive victims of a “brain disease” and thus deserving of social support and medical treatment rather than stigma or moral censure (Leshner 1997)? A more recent view based in learning theory holds that addiction is essentially a powerful habit formed through the accelerated pursuit of valued mental states, not unlike the process of falling in love (Lewis, 2017; see also Earp et al. 2017a; Earp et al. 2017b). Other models have also been proposed.

At first glance, each view seems to imply something different about the kind of person one is when addicted. The weakness-of-will model holds that addiction is a blameworthy matter reflecting imprudent choices or an impoverished character. The brain disease model holds that addiction entails a relatively faultless loss of agency. The learning model holds that addiction reflects ordinary brain functioning taken to an extreme in response to certain patterns and types of reward. Based on these and other differences, it is often assumed that what people think about the qualitative identity of addicted agents will turn on which model of addiction is widely accepted (for a general discussion, see O’Connor and Joffe 2013).

However, empirical support for this view is limited. In particular, the idea that characterizing addiction as a brain disease will combat stigma or reduce attributions of moral responsibility has not been consistently supported (Meurk et al. 2014; Piras et al. 2016; Racine et al. 2017). Moreover, the studies that do exist in this vein tend to treat addiction as a static state, comparing lay attitudes as a function of various ways addiction might be described. But addiction is not something a person is born with. Rather, it is a state one enters into and potentially leaves, in the context of social judgments and identity descriptions that often have a more narrative structure, tracking changes in personal attributes through time (Buchman and Reiner 2009). Given that work in psychology and experimental philosophy has looked at such judgments as they relate to qualitative identity shifts in other contexts, it may be fruitful to apply a similar set of methods to the topic of addiction.

As discussed, this work shows that changes in moral attributes are more important for judgments of altered identity than other personal attributes (Strohminger and Nichols 2014; Strohminger, Knobe, and Newman 2017; Heiphetz, Strohminger, and Young 2017), and that moral deterioration, compared to moral improvement, is especially important for shaping such judgments (Tobia 2015, 2016, 2017). Since addiction is a highly moralized phenomenon, it stands to reason that similar judgments would apply. In other words, insofar as an agent’s becoming addicted to drugs may lead to the perception that they are a “different person”—as suggested by the anecdotes in the previous section—this may be due to a presumed diminishment in moral character that such addiction stereotypically brings about.

To test this hypothesis, we conducted six studies with a combined sample of 3,620 U.S. participants. In Study 1, we sought to establish the basic phenomenon to be explained: Going beyond anecdotes, do people really regard others as undergoing a change in identity as a consequence of addiction? By describing a character named Jim either becoming addicted to drugs or recovering from addiction, and asking participants to rate the extent to which he has changed or stayed the same as a person, we find that addicted Jim is judged to be far closer to a “completely different” person than “completely the same” person as he was before the addiction. In Study 2, which includes a replication of Study 1, we attempt to tease apart whether it is the physical effects of the drug of addiction or the moral implications of taking it that are responsible for such judgments. To do this, we manipulated the moral status of the drug while holding its physical effects constant, describing it as medicine in one condition (good drug) and as an addictive drug (bad drug) in another. We find some support for the “moral status” interpretation, but not without ambiguity. In Study 3, therefore, we made the moral effects of the drug on Jim’s character explicit, finding that moral deterioration led to greater judgments of changed identity than moral improvement, supporting our explanatory framework. To ensure that this was not a vignette-specific effect, in Study 4, we conducted a preregistered conceptual replication and extension study involving four new vignettes, all of which described a character undergoing moral improvement versus deterioration as a result of drug use, with similar results. We also directly asked participants in this study about the extent to which each character had grown closer to, or farther away from, their “true self” as a result of the moral change, finding further support for our hypothesis. In Studies 5 and 6, we consider competing explanations for our findings, and attempt to rule these out. In the end, we find that the moral badness of changes to character associated with drug abuse and addiction is largely responsible for participant intuitions concerning altered identity, corresponding to judgments that the addicted agent has moved away from their good true self.

## Study 1

Study 1 sought to determine whether or to what extent people believe that acquiring an addiction can result in changes to identity. This study and the ones described later were considered exempt by the Institutional Review Board (IRB) at Yale University (IRB Protocol 0907005485). All of the studies conducted for this research project are reported in this article; we affirm that there is no file drawer to skew the reported findings (Rosenthal 1979). All of the materials, data, and syntax for reproducing analyses are available on the Open Science Framework (OSF) at osf.io/bm96x.

### Method

**Participants.** Two hundred eighty-nine U.S. participants were recruited via the online service Mechanical Turk (MTurk), and received $0.40 for their time.^1^ No a priori power analysis was conducted for this preliminary study; sample size was determined by available resources and past experience with experiments of this kind. A post hoc power analysis using G*Power (Faul et al. 2007) with α = .05 revealed that we had 92.9% observed power to detect an effect size of Cohen’s *d* = .45. Participants (*n* = 55) were excluded for failing to complete the entire survey or giving the incorrect answer to an embedded manipulation/attention check. Excluding these participants resulted in a final sample of 235 participants (105 female; *M_age_* = 36.56, *SD* = 11.63).

**Procedure.** Participants completed an online survey in a between-subjects design. Participants were given one of two stories about a man named Jim. In one story, Jim was described as becoming addicted to drugs, and in the other, as recovering from addiction. To stimulate concrete intuitions about the cases presented rather than abstract reasoning about the general relationship between addiction and identity change, specific but morally neutral details were included about Jim and his life. This introductory paragraph read as follows:

Jim is 27 years old. He graduated from Briarcrest High School in a town called Bloomington when he was 17. Since then, he’s attended community college, traveled some, worked different jobs, and learned how to play the guitar. He likes listening to music and spending time with his friends. Jim’s mother is a librarian, and his father works for an insurance company. He has a sister named Mary, and a brother named Albert.

Then participants saw one of two paragraphs (the labels below are for clarity; they were not shown to participants):

*Starting addiction*. Jim didn’t used to be addicted to drugs, but now he is. About a year ago, some big changes happened in Jim’s life, and he became addicted to drugs. Like most addicts, he finds it very difficult to refrain from seeking out and consuming drugs, even when there are bad consequences. When he is prevented from taking his drug, he experiences very unpleasant feelings of withdrawal. He now spends a lot of his time thinking about, and seeking, the drug of his addiction.

*Stopping addiction*. Jim used to be addicted to drugs, but now he isn’t. About a year ago, some big changes happened in Jim’s life, and he stopped being addicted to drugs. Like most addicts, he used to find it very difficult to refrain from seeking out and consuming drugs, even when there were bad consequences. When he was prevented from taking his drug, he experienced very unpleasant feelings of withdrawal. He used to spend a lot of his time thinking about, and seeking, the drug of his addiction.

Participants were asked to “Think about how Jim is right now, compared to how he was before those big changes happened in his life. To what extent do you feel that Jim, as a person, has changed or stayed the same? On the next few pages you’ll receive some questions and statements to try to get at your intuition.” Participants then answered the identity change questions described in the following. As an exploratory measure, they were also asked which model of addiction they personally subscribed to: the “brain disease” (medical) model, or the “weakness-of-will” (moral) model; results for this measure are reported in in the online supplementary materials. They then responded to an attention check and filled out some basic demographic information. At the end of the survey they were debriefed and thanked for their time.

**Measures.*Identity change.*** Participants were given, in random order, two questions and two statements designed to capture their intuitions about whether or to what extent Jim had changed as a person. The questions were:

1. “In terms of changing or staying the same, how much would you say that Jim is the same or a completely different person than before?”
2. “How much has Jim changed as a person, if at all?”

For the first question, participants were given a sliding scale ranging from 0 to 100, with 0 labeled “exactly the same person as before” and 100 labeled “completely different person than before,” and were asked to mark their response anywhere along the scale. For the second question, the scale ranged from 0 (“not at all”) to 100 “(a great deal”). The statements were:

1. “There is a sense in which Jim is not really the same person anymore.”
2. “Jim is now pretty different from what he used to be all about.”

For each statement, participants were asked to indicate how much they agreed or disagreed along the same 100-point scale, ranging from 0 (“completely disagree”) to 100 (“completely agree”). The four items formed a reliable measure (α = .926), *identity change*, that served as the dependent variable.

***Manipulation/attention check*.** Participants were told, “This is the last question, just to check if you remember the story about Jim. At the end of the story, was Jim addicted to drugs or not addicted to drugs?” They were then asked to pick between (1) “Jim was addicted to drugs” and (2) “Jim was NOT addicted to drugs.” Participants who chose the incorrect answer based on their condition were excluded from all further analyses.

### Results

As predicted, there was a main effect of condition, such that Jim was perceived as undergoing more identity change when becoming addicted (*M* = 74.40, *SD* = 19.63) compared to recovering from addiction (*M* = 65.05, *SD* = 21.91), *t*(233) = 3.45, *p* = .001, *d* = .45. In both conditions, the mean was above the midpoint of the scale (becoming addicted: *t*(122) = 13.79, *p* < .001, *d =* 1.76; recovering from addiction: *t*(111) = 7.27, *p* < .001, *d* = .97), suggesting that, in either case, Jim was perceived as closer to a “completely different” person than “completely the same” person after the described changes.

### Discussion

Study 1 was designed to answer the question, “Do people regard others as undergoing a change in identity as a consequence of becoming addicted?” Our findings point to a positive answer. Based on only a minimal description of becoming addicted to drugs, participants rated Jim as highly changed as a person compared to how he was before the addiction. Curiously, however, participants also rated Jim as highly changed when *recovering* from addiction (albeit to a lesser degree). How might one explain this finding?^2^

Imagine that Jim is your friend. For quite some time, you have known him as someone addicted to drugs. As described in the vignette, he always found it difficult to refrain from using drugs, even when there were bad consequences (a core sign of addiction on many accounts). He also showed symptoms of physiological dependence, like withdrawal, and in general his thoughts and behavior were consumed with seeking out his next hit. So, however disagreeable some of these attributes might be from a certain perspective, that is the Jim you know. But now, some “big changes” have happened in his life, and those familiar attributes no longer apply. Jim has cleaned up his act, to be sure, and that is presumably a good thing—but he does seem rather different now compared to how he was before. Thus, the observed ratings for identity change in this condition should not be too surprising.

What is important for our purposes, however, is the difference between conditions, and in particular the degree of change between starting and stopping addiction. This difference was in the expected direction: Participants rated Jim as less changed as a person when he recovered from his addiction to drugs (an improvement) than when he became addicted to drugs (the reverse). Thus, the overall pattern of results is consistent with the so-called Phineas Gage Effect (Tobia 2015), according to which a person is seen as undergoing greater identity change if they experience a moral deterioration in their character, as compared to a moral improvement.

There is a catch, however. The vignettes don’t actually say that Jim’s moral characteristics changed from Time 1 to Time 2, apart from a passing reference to his seeking out and consuming drugs “even when there were bad consequences.” Instead, they primarily refer to various physical or behavioral effects that are often associated with drug addiction, namely, finding it hard to refrain from taking the drug, experiencing unpleasant feelings when one is prevented from taking it, and so on.

One possibility, then, is that participants were tracking this physical-behavioral dimension, with the intuition being that losing such unpleasant aspects of addiction—presumably caused by the drug itself—is not as disruptive to identity as acquiring them. After all, if one starts to take a drug that causes one to be distracted and unhappy when one is not on it, it may seem that it is the drug that is really doing the work. By contrast, if one stops taking such a drug, one might be seen as simply reverting to one’s baseline self. This could explain why Jim was seen as less changed as a person when he lost his addiction compared to acquiring it, without the need to invoke moral considerations. To explore this issue, we conducted another study.

## Study 2

Our goal in Study 2 was to keep the physical consequences of starting or stopping the use of a drug constant, while manipulating the moral valence of such use. One way to keep the physical effects constant while changing moral valence is to describe a drug as “medicine” in one condition (where it will presumably be seen as morally good or at least neutral) while describing it as “addictive” in another condition (where it will presumably be seen as morally bad, given the context and framing), keeping everything else the same. This is the approach we took in Study 2.

### Method

Participants. Four hundred and fifty U.S. participants were recruited via MTurk, and received $0.40 for their time. A post hoc power analysis with α = .05 revealed that we had 99.9% power to detect an effect size of Cohen’s *f* = .25. Participants (*n* = 34) were excluded from analyses for failing the manipulation check or not finishing the survey. Excluding these participants resulted in a final sample of 416 participants (162 female; *M_age_* = 35.25, *SD* = 11.52).^3^

Procedure. This study had a 2 (drug use: starting, stopping) by 2 (drug valence: good, bad) between-subjects experimental design. Participants read one of four stories: two in which Jim was described as either becoming addicted to drugs or recovering from his addiction to drugs (the same stories as in Study 1), and two in which he was described as either becoming addicted to medication or recovering from his addiction to medication. All physical effects of the “addictive drugs” and “medication” were held constant across conditions. The same introductory paragraph from Study 1 was used, as was the four-item identity change measure (α = .927). Complete materials can be found in the supplementary materials.

### Results

A 2 × 2 analysis of variance (ANOVA) with the preceding design was conducted on identity change. There was a main effect of drug valence on identity change, *F*(1, 412) = 26.347, *p* < .001, η_p_^2^ = .060, with bad drugs resulting in greater perceived identity change (*M* = 66.53, *SD* = 22.55) than good drugs (*M* = 55.41, *SD* = 21.93). There was no main effect of condition, however, and no interaction (*p*s > .417).

### Discussion

In Study 1, Jim was seen as changing more as a person when he started taking a “bad” drug and became addicted, than when he stopped taking the drug and recovered from his addiction. Unexpectedly, in Study 2, this effect did not replicate. In fact, the mean score for identity change in the starting/bad condition (*M* = 66.74, *SD* = 24.92) is quite similar to the mean in the stopping/bad condition (*M* = 66.37, *SD* = 20.61), even though these conditions are identical to the ones from Study 1. This raises the possibility that our initial finding from Study 1 was a fluke or statistical artifact. Before going any further, then, we decided to run an exact replication of Study 1, albeit with a larger sample size and without the exploratory question concerning models of addiction.^4^

Results were mixed. Consistent with Study 1, participants in the replication study did see Jim as changing more as a person in the starting/bad condition (*M* = 70.93, *SD* = 20.36) than in the stopping/bad condition (*M* = 67.35, *SD* = 17.49), *t*(327) = 1.67, *p* = .096, *d* = .19. However, in contrast to Study 1, the difference in means was only marginal by conventional standards of statistical significance, and the observed effect size was much smaller (*d* = .19 in the replication vs. *d* = .45 in the original). The implications of this outcome are unclear. Particularly odd is the near equality of the starting/bad versus stopping/bad means in Study 2, compared to the large and statistically significant difference between these means in Study 1, and the marginal difference observed in the replication. However, given the main effect of drug valence in Study 2, the emerging picture seems to be that if starting versus stopping a drug does play a role in shaping intuitions about identity change, it is a smaller role than that played by the goodness or badness of the drug, regardless of whether one is starting or stopping its use. To explore this issue, we conducted a follow-up study.

## Study 3

In Study 3, we sought to shift the focus more definitively from the physical effects of drug use to its moral effects. Recall that, in Study 2, we still did not explicitly state how Jim’s moral character was changed by the addictive drug, regardless of how it was described. Rather, we retained the physical description of the drug from Study 1, while attempting to manipulate the moral status of its use by characterizing it as medication in one set of conditions. Our assumption was that participants would see it as permissible and even desirable—in short, good—to use a drug if it is serving a medical purpose, whereas they would see it as bad to use a drug with similar addictive properties if it was not serving a medical purpose. However, given increased public attention to the crisis surrounding addiction to prescription opioids—see the introduction—this distinction may not have been as strong as we assumed. In Study 3, therefore, we decided to make explicit the moral effects of starting or stopping the use of a drug, either medical or recreational, on Jim’s personal characteristics and behavior. In one set of conditions, Jim now experiences clear moral improvement as a result of starting or stopping the use of a drug, while in the other set, he experiences clear moral deterioration.

In order to describe such moral changes without being too heavy-handed (that is, without explicitly stating that Jim’s moral character has changed as such, or asking participants to rate Jim on his moral character directly), it seemed necessary to give participants a fuller description of his baseline attributes. In the previous studies, this baseline was nondescript: Participants were told Jim’s age, the fact that he likes listening to music, the occupations of his parents, and so forth. For this study, however, we added a distinctive quality to the introductory paragraph, as follows:

Jim is 27 years old. He graduated from Briarcrest High School in a town called Bloomington when he was 17. Since then, he’s attended community college, traveled some, worked different jobs, and learned how to play the guitar. Most importantly of all though, since he was a little kid, Jim’s biggest dream has been to become a successful poet.

Then, for each of the four conditions—starting or stopping use of a recreational drug; starting or stopping use of medication—we made explicit some of Jim’s moral qualities at Times 1 and 2 (i.e., his motivation, responsibility, goal commitment, and reliability as a friend), so that participants could infer the relevant change in moral character. All other aspects of the procedure, materials, and analysis were kept the same as in Study 2.

### Method

**Participants.** Six hundred and four U.S. participants were recruited via MTurk, and received $0.50 for their time. Sample size was determined by setting the floor at 450 participants to match the previous study, with the ceiling set by available funding. A post hoc power analysis with α = .05 revealed that we had 99.4% power to detect an effect size of Cohen’s *f* = .19 for the predicted interaction. Participants (*n* = 25) were excluded from analysis for failing the attention check or not finishing the survey. Excluding these participants resulted in a final sample of 579 participants (280 female; *M_age_* = 37.34, *SD* = 12.20).

**Procedure.** This study had a 2 (drug use: starting, stopping) by 2 (drug valence: good, bad) between-subjects experimental design. The procedure was the same as in Study 2, with the same four-item identity change measure (α = .908). Complete materials can be found in the supplementary materials.

### Results

**Figure 1.**
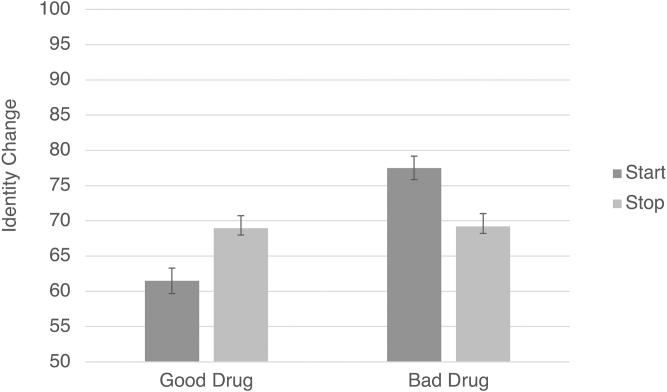
Study 3 results: the effects of starting versus stopping a good or bad drug on judgments of identity change. Error bars represent standard error; the Y axis has been truncated for ease of interpretation.

A 2 × 2 ANOVA with the preceding design was conducted on identity change. Consistent with Study 2, although there was no main effect of starting/stopping (*p* = .821), there was a significant main effect of drug valence, *F*(1, 575) = 21.45, *p* < .001, η_p_^2^ = .036, with bad drugs resulting in greater perceived identity change (*M* = 73.71, *SD* = 19.29) than good drugs (*M* = 65.39, *SD* = 23.45). However, this time, the effect was qualified by a significant interaction between drug valence and condition: *F*(1, 575) = 20.168, *p* < .001, η_p_^2^ = .034. To break this interaction down, we conducted two separate independent-samples *t*-tests. In the good drug conditions, there was greater perceived identity change when Jim stopped taking the drug, leading to moral deterioration (*M* = 68.99, *SD =* 21.64), than when he started taking the drug, leading to moral improvement (*M* = 61.50, *SD* = 24.75), *t*(279) = −2.71, *p* = .007, *d* = .32. Meanwhile, in the bad drug conditions, there was greater perceived identity change when Jim started taking the drug, leading to moral deterioration (*M* = 77.52, *SD =* 17.82), than when he stopped taking the drug, leading to moral improvement (*M* = 66.23, *SD =* 20.04), *t*(296) = 3.78, *p* < .001, *d* = .44 (Figure 1).

### Discussion

Several interesting findings emerged from this study. First, it replicates the main effect of drug valence (good versus bad) from Study 2: that is, independent of whether Jim starts or stops using a drug, there is a main effect of greater perceived identity change when the drug in question is bad compared to good. This might suggest that being “mixed up” in the putatively immoral world of illegal, recreational drugs—even if Jim has recently extricated himself—is enough to drive perceptions of greater identity change compared to being “mixed up” in the putatively good, or at least less bad, world of prescription medication.

Second, there was the predicted interaction: Jim’s identity was judged to have changed more when he experienced moral deterioration (whether that was caused by starting a bad drug or stopping a good drug) than when he experienced moral improvement (whether that was caused by starting a good drug or stopping a bad drug). This finding brings the evidence more into line with our proposed theoretical framework—the good-true-self framework—according to which greater perceived disruption to identity should occur when an agent becomes morally worse compared to morally better.

Finally, there was no main effect of starting versus stopping. In other words, simply starting to take a drug of one kind or another (whether medical or recreational) was seen as no more relevant to identity change than stopping such drug use altogether. This might suggest that the supposed unnaturalness of being addicted to an “artificial” chemical substance, as in the case of various drug addictions, is not a major factor in explaining why people seem so different when grappling with an addiction compared to not. Indeed, even when Jim had to start taking a drug in order to experience moral improvement (and live out his dream of becoming a poet), he was seen as less changed as a person than when he stopped taking the drug (i.e., “went off his meds”) insofar as this led to moral deterioration. It is thus the good/bad dimension (whether of drug type or change in moral character) that seems primarily responsible for driving participant intuitions about the degree of change in Jim’s identity. Specifically, when the drug or direction of change is good, holding magnitude and means of change constant, Jim does not seem so different as a person compared to when the drug or direction of change is bad.

Taken together, the results presented in this study provide the strongest empirical support for our theoretical expectations. It would be concerning, then, if they turned out to be due to some idiosyncratic feature of Jim and his poetic ambitions. To address this issue, and in light of ongoing concerns about replicability in psychology and experimental philosophy (Earp and Trafimow 2015; Cova et al. 2018; LeBel et al. 2018), we decided to conduct a preregistered replication and extension study, in which we presented participants (between subjects) with four structurally similar vignettes—in addition to the one about Jim—describing other characters with a wide range of personal attributes and goals, but all with the shared feature of experiencing moral improvement versus deterioration as a result of starting or stopping the use of a drug.

## Study 4

To confirm the results of Study 3, and to ensure that they were not vignette specific but rather reflective of a deeper pattern of moral intuition, we conducted a preregistered replication study, adding four new structurally similar vignettes. In addition to Jim the Poet, these vignettes concerned Lisa the Science Teacher, Amal the Chiropractor, Jasmine the Artist, and Dale the Truck Driver. The full text of these vignettes can be seen in the online supplementary materials, and the time-stamped preregistration form can be accessed at http://aspredicted.org/blind.php?x=ri4c2q.

For this study, our primary dependent measure was the same as in all previous studies, namely, identity change. However, we decided to add two additional measures for purposes of exploratory analysis: one asking about changes to the character’s true self, to see whether a more direct question about the theoretical construct of interest would show results similar to those of the relatively indirect measure we had so far been using; and one asking about the character’s responsibility for their behavior while taking the drug in question. The reason we added the latter question was to determine whether judgments of identity change might simply be tracking perceived responsibility. We also reincorporated the addiction model question from Study 1 based on its theorized relationship to such responsibility judgments. The results for these last two measures are reported in the supplementary materials, but in brief we found that identity judgments did not simply reduce to responsibility judgments, and that the relationship between such judgments and participants’ preferred model of addiction was—in contrast to what is commonly hypothesized, as described in the introduction—negligible.

### Method

**Participants.** An a priori power analysis using G*Power 3 (Faul et al. 2007) revealed that for each vignette, a sample size of *n* = 225 would be required to detect a small-to-medium effect size using a conventional α of .05 with .80 power. With five vignettes, this yields a total desired sample size of *n* = 1125. Ultimately, 1,342 U.S. participants took the survey via MTurk, each receiving $0.50 for their time. Participants were excluded from analysis for failing the attention check (*n* = 121) or not finishing the survey (*n* = 43). Excluding these participants resulted in a final sample of 1,178 participants (551 female; *M_age_* = 36.13, *SD* = 11.29).

**Procedure.** This study had a 2 (drug use: starting, stopping) by 2 (drug valence: good, bad) by 5 (vignette: Jim, Lisa, Amal, Jasmine, Dale) between-subjects experimental design. The procedure was the same as in Study 3, with the same four-item identity change measure (α = .920) and a new, single-item true self measure. Complete materials can be found in the supplementary materials.

### Results

**Confirmatory analysis. *Identity change.*** A 2 × 2 × 5 ANOVA with the described design was conducted on identity change. Importantly, there was no main effect of character (*p* = .093), nor were there interactions between character and condition (*p* = .854) or drug valence (*p* = .390), nor interactions among character, condition, and drug valence (*p* = .073). Thus, no single vignette—such as the story about Jim used in the previous study—was responsible for driving the results reported in this section.

As with Studies 2 and 3, there was a significant main effect of drug valence on judgments of identity change, *F*(1, 1158) = 13.24, *p* < .001, η_p_^2^ = .011, with bad drugs resulting in greater perceived identity change (*M* = 72.87, *SD* = 20.97) than good drugs (*M* = 68.41, *SD* = 21.38), replicating our main finding. Also consistent with Studies 2 and 3, there was no main effect of starting/stopping (*p* = .804). The predicted interaction also obtained. Just as in Study 3, there was a significant interaction between drug valence and condition: *F*(1, 1158) = 55.48, *p* < .001, η_p_^2^ = .046, which we decomposed by performing two separate 2 (condition) by 5 (character) ANOVAs.

**Figure 2.**
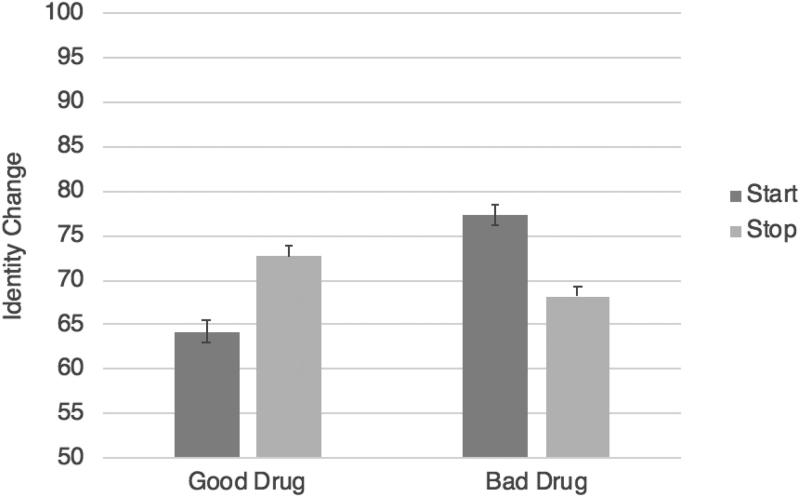
Study 4 results (identity change): the effects of starting versus stopping a good or bad drug on judgments of identity change. Error bars represent standard error; the Y axis has been truncated for ease of interpretation.

As predicted, in the good drug conditions, when the character started taking a good drug, leading to moral improvement, their identity was seen as changing relatively less (*M* = 64.18, *SD* = 21.68) than when they stopped taking a good drug, leading to moral deterioration (*M* = 72.67, *SD* = 20.24), *F*(1,534) = 23.15, *p* < .001, η_p_^2^ = .042. Also as predicted, in the bad drug conditions, the opposite pattern obtained. In other words, when the character started taking a bad drug, leading to moral deterioration, their identity was seen as changing relatively more (*M* = 77.37, *SD* = 19.93) than when they stopped taking a bad drug, leading to moral improvement (*M* = 68.13, *SD* = 21.03), *F*(1,624) = 33.25, *p* < .001, η_p_^2^ = .051 (Figure 2).^5^

**Exploratory analysis. *True self.*** As preregistered, an exploratory 2 × 2 × 5 ANOVA with the described design was conducted on true self judgments. As with identity change, there was a significant main effect of drug valence on true self judgments, *F*(1,1156) = 5.52, *p* = .019, η_p_^2^ = .05, with bad drugs resulting in greater judged distance away from one’s true self (*M* = 54.65, *SD* = 35.12) than good drugs (*M* = 50.83, *SD* = 30.91). Departing from the pattern of results for identity change, there was also a main effect of condition (starting, stopping), *F*(1,1156) = 94.98, *p* < .001, η_p_^2^ = .08, with starting any kind of drug resulting in greater judged distance away from one’s true self (*M* = 61.39, *SD* = 32.18) than stopping any kind of drug (*M* = 44.11, *SD* = 32.12). There was no main effect of character (*p* = .333), nor were there interactions between character and condition (*p* = .657) or drug valence (*p* = .935).^6^

**Figure 3.**
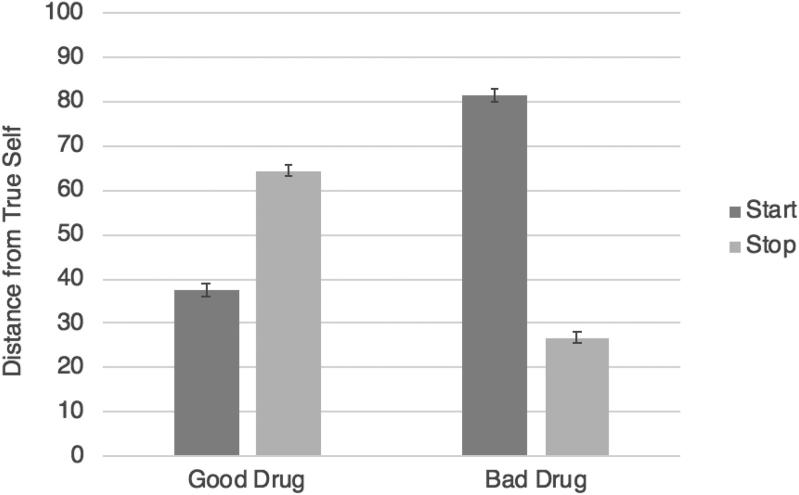
Study 4 results (true self): the effects of starting versus stopping a good or bad drug on judgments of distance from the true self. Error bars represent standard error.

With respect to our main hypothesis, the predicted interaction between drug valence and condition obtained: *F*(1, 1156) = 757.95, *p* < .001, η_p_^2^ = .40. To decompose this interaction, we performed two separate 2 (condition) by 5 (character) ANOVAs. In the good drug conditions, when the character started taking a good drug, leading to moral improvement, they were seen as being far closer to their true self (*M* = 37.63, *SD* = 27.42) than when they stopped taking a good drug, leading to moral deterioration (*M* = 64.07, *SD* = 28.50), *F*(1, 533) = 116.66, *p* < .001, η_p_^2^ = .180.^7^ In the bad drug conditions, the opposite pattern obtained: When the character started taking a bad drug, leading to moral deterioration, they were seen as being much further away from their true self (*M* = 81.27, *SD* = 20.16) than when they stopped taking a bad drug, leading to moral improvement (*M* = 26.55, *SD* = 23.82), *F*(1, 623) = 966.13, *p* < .001, η_p_^2^ = .608 (Figure 3).

**Figure 4.**
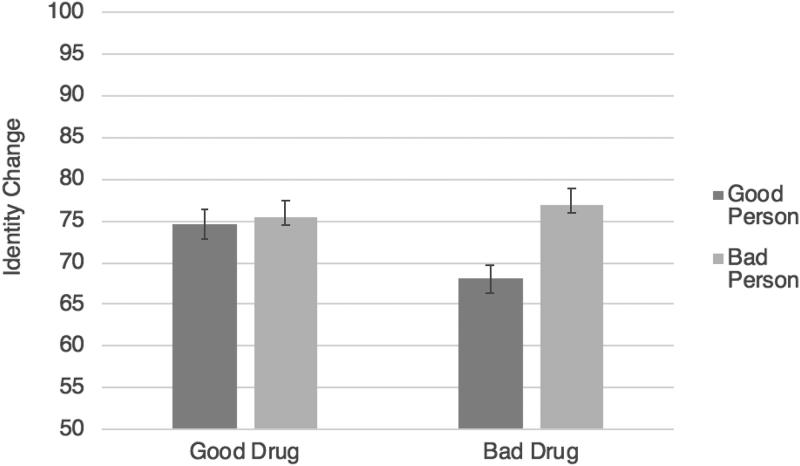
Study 5 results (identity change): the effects of taking a good or bad drug leading to becoming a good or bad person on judgments of identity change. Error bars represent standard error; the Y axis has been truncated for ease of interpretation.

**Figure 5.**
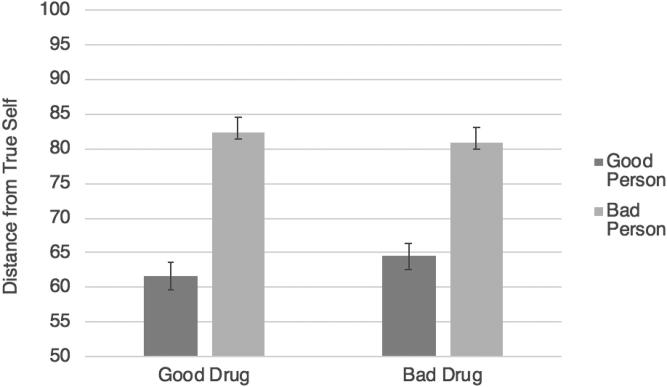
Study 5 results (true self): the effects of taking a good or bad drug leading to becoming a good or bad person on judgments of distance from the true self. Error bars represent standard error; the Y axis has been truncated for ease of interpretation.

**Figure 6.**
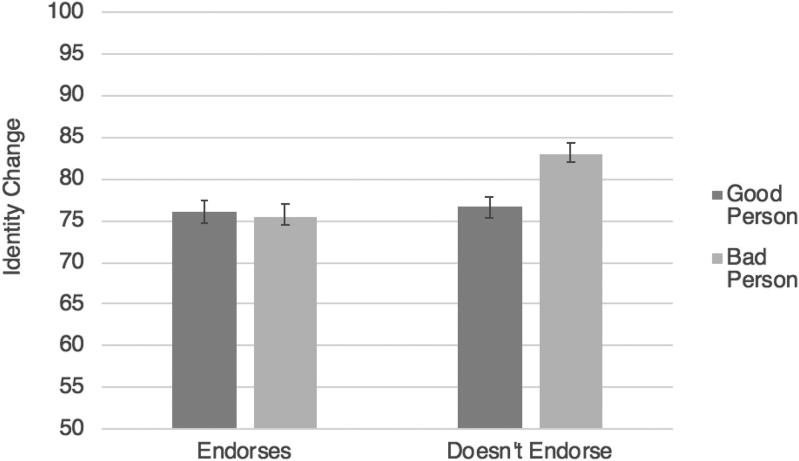
Study 6 results (identity change): the effects of Jim endorsing or not endorsing becoming a good or bad person on judgments of identity change. Error bars represent standard error; the Y axis has been truncated for ease of interpretation.

**Figure 7.**
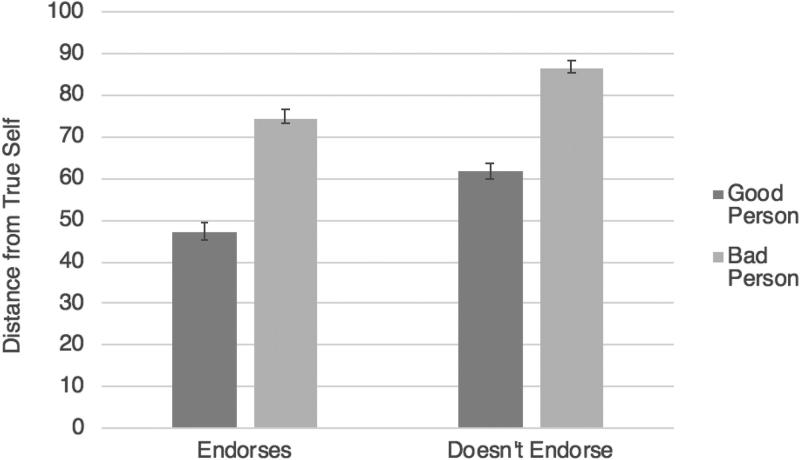
Study 6 results (true self): the effects of Jim endorsing or not endorsing becoming a good or bad person on judgments of distance from the true self. Error bars represent standard error.

### Discussion

Taken together, the results from Studies 1 through 4 appear to support the good-true-self theoretical framework we introduced at the beginning of this article. But there are some remaining ambiguities. Specifically, it is hard to tell the relative contribution of goodness or badness of drug versus goodness or badness of change in moral character in affecting participant perceptions of changed identity. This is because the good drug (i.e., medical substance) was described as having good effects on the moral attributes and behavior of the characters, whereas the bad drug (i.e., recreational substance) was described as having bad effects. Because the presumed moral quality of the drug itself is conflated with the direction of change in moral character, the strongest test of the good-true-self theory as it relates to addiction remains elusive: Do people who become addicted to drugs seem like “not the same person” as before because of something about their drug of addiction and how we think of it and its various effects? Or is it the negative moral changes to the addicted person’s character that truly explain the intuition? To address this issue, we conducted a fifth study.

## Study 5

Recall that, according to good-true-self theory, each of us is (as a default) perceived as having a true inner essence that is fundamentally good: The more we move away from this essence, the further we appear to be from our true self. By contrast, when we move toward the essence—by being and acting as moral as we can—the closer we appear to be to our true self, and thus less changed as a person (as measured from that anchor point) over time and across other forms of change. Accordingly, if becoming addicted to a putatively bad, illegal substance nevertheless caused an agent to become more moral—by whatever strange mechanism—people should judge the agent as having undergone less change in identity at Time 2 compared to the case in which she becomes morally worse by one means or another.

To test this idea, we began by eliminating the starting versus stopping distinction, since previous studies showed that this was far less important than moral valence; we also returned to a single story about Jim, since Study 4 showed that using different vignettes did not substantially affect the main findings. This freed us up to create set of cases in which all aspects were held constant apart from the moral valence of the drug and the direction of change in Jim’s moral character, allowing us to better tease those factors apart.^8^

### Method

**Participants.** Six hundred and one U.S. participants were recruited via MTurk, and received $0.50 for their time. Sample size was set to match Study 3. A post hoc power analysis with α = .05 revealed that we had 67.3% power to detect an effect size of Cohen’s *f* = .10 for the predicted interaction. Participants (*n* = 25) were excluded from analysis for failing the attention check or not finishing the survey. Excluding these participants resulted in a final sample of 576 participants (274 female; *M_age_* = 36.24, *SD* = 11.01).

**Procedure.** This study had a 2 (drug valence: good, bad) by 2 (moral character change valence: good, bad) between-subjects experimental design. The procedure was the same as in Study 4, with the same four-item identity change measure (α = .924) and the new, single-item true self measure. After the same initial prompt described in Study 1, participants read one of four stories, in which Jim’s moral character underwent a change for the better or worse as a result of taking a good (medicine) or bad (illegal, recreational) drug. The introductory paragraph described in Study 3 was used for all four conditions, except that the sentence describing Jim’s desire to become a poet was deleted. Complete materials can be found in the supplementary materials.

### Results

***Identity change.*** A 2 × 2 ANOVA with the described design was conducted on identity change. In contrast to previous studies, there was no main effect of drug valence on judgments of identity change (*p* = .164), whereas there was a main effect of moral character change valence, *F*(1,572) = 4.84, *p* = .006, η_p_^2^ = .01, with Jim judged to have undergone less change in his identity when he became a good person (*M* = 71.304, *SD* = 22.52) compared to when he became a bad person (*M* = 76.25, *SD* = 20.74). This result is consistent with our predictions and theoretical framework, suggesting that it is the change in the moral character of the person, rather than the moral characteristics of the drug itself, that is most relevant in affecting intuitions about identity change.

An interaction was observed between drug valence and moral character change valence, *F*(1,572) = 4.84, *p* = .028, η_p_^2^ = .01, which we decomposed with two separate *t*-tests. Curiously, in the good drug conditions, ratings for identity change were similar regardless of whether the drug caused Jim to become a good person (*M* = 74.57, *SD* = 18.63) or a bad person (*M* = 75.53, *SD* = 21.19), *t*(288) = −.41, *p* = .680, *d* = .05. However, in the bad drug conditions, as predicted, participants rated Jim as undergoing less identity change when he became a good person (*M* = 68.04, *SD* = 25.42) compared to when he became a bad person (*M* = 77.00, *SD* = 20.30), *t*(284) = −3.22, *p* = .001, *d* = .39. As can be seen in Figure 4, Jim was rated as having the least identity change in the “counterintuitive”’ case in which a bad drug actually caused him to become a good person. This is exactly what was predicted by our theory.

***True self.*** Next, a 2 × 2 ANOVA with the same design was conducted on true self. Consistent with the identity change analysis, there was no main effect of drug valence on judgments about the true self (*p* = .745), whereas there was a main effect of moral character change valence, *F*(1,572) = 81.63, *p* < .001, η_p_^2^ = .13, with Jim being judged much closer to his true self when he became a good person (*M* = 63.04, *SD* = 27.27) compared to when he became a bad person (*M* = 81.68, *SD* = 20.66). This result, too, is consistent with our predictions and theoretical framework, suggesting that it is the change in the moral character of the person, rather than the moral characteristics of the drug itself, that is most relevant in affecting intuitions about the true self. No interactions were observed (*p* = .281). For the overall pattern of results see Figure 5.

### Discussion

In previous studies, the moral valence of the drug and the moral valence of the change in character were overlapping. To tease these variables apart, in Study 5, we created a scenario in which a bad drug counterintuitively resulted in moral improvement, predicting that this would lead to lower ratings for identity change compared to a drug of whatever valence resulting in moral deterioration. This is what we found, supporting our theory. Surprisingly, however, when a good drug resulted in moral improvement (which should also have led to lower ratings of identity change), Jim was rated as having changed as a person to a similar extent as when a drug of either valence resulted in moral deterioration. This was not predicted by our theory. One possibility is that the anomalous result was due to sampling error. Indeed, when we turn to true self ratings, we see the expected pattern of results: When a good or bad drug leads to moral deterioration, the character is judged as being much further from his true self than when a drug of either valence leads to moral improvement.

We have now provided substantial evidence that the predictions of good-true-self theory (GTS) are borne out in the case of addiction and drug use. In so doing, we have united two previously separate literatures, which we hope will inspire further research. However, we have by no means shown that GTS is the only theory that can explain the results we have observed. Another theory in philosophy that touches on similar questions is Frankfurt’s (1971) account of free will and the concept of a person.^9^ In his famous comparison of two people addicted to narcotics, one willing and the other unwilling, Frankfurt draws a distinction between first-order desires (e.g., the desire to take a drug or refrain from taking a drug) and what he calls second-order volitions: a kind of meta-desire by which a person “identifies” with one first-order desire or another.

On a broadly Frankfurtian theory (FT), one might think that this second-order endorsement is what reveals a person’s true self, not just the moral valence of one’s desires, disposition, or behavior. But then, people do tend to endorse or identify with the positive aspects of their moral character, whereas they tend to resist or disidentify with the negative aspects. Thus, in the typical case, GTS and FT will make the same prediction. Specifically, moral deterioration could be seen as (1) movement away from one’s good true self, which would lead to higher ratings for identity change, or (2) movement away from what one identifies with in terms of second-order volitions, which would also lead to higher ratings. It is only in the “counterintuitive” case where one actually endorses negative changes to one’s moral character that the theories come apart: FT predicts relatively low ratings for perceived identity change, whereas GTS predicts relatively high ratings. To address this issue, we conducted one final study, which we preregistered with aspredicted.org in order to minimize researcher degrees of freedom (http://aspredicted.org/blind.php?x=8fg5kf).

## Study 6

### Method

**Participants.** A conservative a priori power analysis using G*Power 3 revealed that a sample size of *N* = 787 would be required to detect a small effect size using a conventional α of .05 with .80 power. To account for possible exclusions, we recruited 800 participants on MTurk; 798 completed the entire survey and passed a simple test designed to catch any automated bots, which had become a concern in the interval between running the previous studies and the current study (Dreyfuss 2018). Participants received $0.50 each for their time. Following the preregistration, participants were excluded prior to data analysis for failing one or both of two embedded attention checks (*N* = 162). This resulted in a final sample of 636 participants (351 female; *M_age_* = 37.94, *SD* = 12.44).

**Procedure.** This study had a 2 (moral character change valence: good, bad) by 2 (second-order endorsement: yes, no) between-subjects experimental design. The procedure was the same as in Study 5, with the same four-item identity change measure (α = .874) and the new, single-item true self measure. Participants were given the same initial prompt described in Study 1. They then saw one of four stories, in which Jim’s moral character underwent a change for the better or worse as a result of taking a drug (described as medication in all four cases), but in which he had second-order volition to be either a “bad boy” or a “good old Jim” before undergoing the change. The introductory paragraph described in Study 5 was used for all four conditions. Complete materials can be found online (https://osf.io/bm96x/).

### Results

**Confirmatory analysis. *Identity change.*** A 2 × 2 ANOVA with the previously described design was conducted on identity change. As predicted by GTS, there was a main effect of moral-character-change valence on judgments of identity change, *F*(1,632) = 4.40, *p* = .036, η_p_^2^ = .01, with Jim judged to have undergone less change in his identity when he became a good person (*M* = 76.34, *SD* = 18.88) compared to when he became a bad person (*M* = 79.81, *SD* = 15.81), independent of whether he actually endorsed the moral change from a second-order perspective. And as predicted by FT, there was a main effect of endorsement, *F*(1,632) = 8.48, *p* = .004, η_p_^2^ = .01, with Jim judged to have undergone less change in his identity when he endorsed the moral change (*M* = 75.77, *SD* = 16.23) compared to when he did not endorse the moral change (*M* = 79.65, *SD* = 18.47), independent of whether he became a good or bad person.

An interaction was also observed, *F*(1,632) = 6.08, *p* = .014, η_p_^2^ = .01, which was decomposed with two separate *t*-tests. Looking just at the cases where Jim endorsed the moral change, it made no difference whether the change was good (*M* = 76.00, *SD* = 16.27) or bad (*M* = 75.49, *SD* = 16.24), *t*(279) = .26, *p* = .793, *d* = .03. When Jim did not endorse the moral change, however, he was judged to have undergone far less change in his identity when he became a good person (*M* = 76.62, *SD* = 20.88) compared to a bad person (*M* = 82.98, *SD* = 14.75), *t*(353) = −3.29, *p* = .001, *d* = .35, consistent with GTS (Figure 6).

**Exploratory analysis. *True self.*** A 2 × 2 ANOVA with the same design was conducted on true self. Consistent with the identity change analysis, there was a main effect of moral-character-change valence on judgments about the true self, *F*(1,632) = 163.34, *p* < .001, η_p_^2^ = .21, with Jim judged to be much closer to his true self when he became a good person (*M* = 55.18, *SD* = 29.70) compared to when he became a bad person (*M* = 81.25, *SD* = 20.78), independent of whether he actually endorsed the moral change from a second-order perspective. There was also a main effect of endorsement, *F*(1,632) = 43.73, *p* = .001, η_p_^2^ = .07, with Jim judged to be much closer to his true self when he endorsed the moral change (*M* = 59.19, *SD* = 30.56) compared to when he did not endorse the moral change (*M* = 73.52, *SD* = 26.12), independent of whether he became a good or bad person. This time there was no interaction (*p* = .537). See Figure 7.

### Discussion

Results from Study 6 suggest that both GTS and FT have independent explanatory power in predicting judgments of identity change following changes in moral character as a consequence of drug use. However, when it comes to judgments of distance from the true self, the main effect of moral-character-change valence (η_p_^2^ = .21) is fully three times greater than the effect of second-order endorsement (η_p_^2^ = .07), suggesting that GTS has certain advantages over FT in explaining participant intuitions about such cases. Finally, in the “counterintuitive” case in which the agent actually endorses negative changes in moral character, participants judged far greater distance from the true self compared to when the agent endorsed positive changes, contrary to the prediction of FT but consistent with that of GTS.

## General discussion

In this article, we sought to extend recent work in psychology and experimental philosophy to a perennial issue in bioethics, namely, the relationship between addiction and identity. But rather than focusing on judgments about qualitative identity, as is typical for such discussions, we focused on judgments of identity persistence: the extent to which an individual is seen as the same person despite having undergone a transformative experience.

In Study 1, we found that U.S. participants rated a character who became addicted to drugs as far closer to “a completely different person” than “completely the same person” as he was before becoming addicted. In Study 2, to see whether it was the moral or physical aspects of the drug that were responsible for this effect, we described the drug as medicine in one condition (good drug), and as an addictive drug (bad drug) in another, finding that the bad drug led to higher ratings for identity change. In Study 3, we made explicit the effects of addiction on moral character to narrow in on the explanatory framework outlined in the introduction, finding that negative changes to moral character led to higher ratings for identity change, as predicted.

In Study 4, we ruled out vignette-specific effects by conducting a preregistered replication and extension in which five different characters underwent moral improvement versus deterioration as a result of starting or stopping the use of a drug. In this study we again found that moral deterioration led to increased ratings for identity change, as well as increased ratings of distance from the true self, as we had predicted. In Study 5, we decoupled the moral valence of the drug from the moral valence of the change in character, to resolve a potential confound. In the critical test case in which a bad drug actually led to moral improvement, the character’s identity was judged as having changed the least, consistent with GTS. Finally, we considered a competing explanation for our findings based on the work of Frankfurt (1971). Although second-order endorsement of one’s moral character did reduce judgments of identity change and distance from the true self compared to the lack of such endorsement, as predicted by FT, we still showed independent effects of moral valence of character change as predicted by GTS. And for distance from the true self, the direction of moral change in character had a much bigger effect on participant judgments about identity change than did second-order endorsement, further supporting GTS.

## Identity change—number or quality?

There are several questions left open by our findings. One concerns the concept of identity at play in the ratings gathered throughout the six studies. Specifically, when someone is judged to be an “entirely different person” after becoming addicted to drugs—as illustrated by the anecdotes at the beginning of this article—we must ask: In what sense are they seen as not the same person?

Starmans and Bloom (2018) have recently argued that much of the current literature on identity change, including the seminal article by Strohminger and Nichols (2014), has been insufficiently clear about the sense of identity being invoked. To understand such expressions as “my son is not the same person anymore now that he is addicted to drugs,” these authors argue, a conceptual distinction must be drawn between changes in numerical identity and changes in qualitative identity.

Numerical identity refers to a single entity persisting over time, as when baby Jim is identical to adult Jim. Thus, if you tickled baby Jim, and later tickled adult Jim, you have tickled the same person twice (Starmans and Bloom 2018). Qualitative identity, by contrast, refers to the sharing of essential properties: If Jim and his twin John are exactly alike in terms of fundamental personality (and other) characteristics, they may be qualitatively identical—that is, extremely or even perfectly similar—but they are not numerically identical. So, for example, it would be mistaken and morally wrong to arrest John for a crime that Jim committed.^10^

Similarly, if pre-addiction Jim and post-addiction Jim are sufficiently different from one another in terms of fundamental personality characteristics, we might say, “Jim is not the same person anymore,” but—according to Starmans and Bloom—this should be understood as a figure of speech: “a way of saying that there has been significant psychological change, not that one person has [literally] ceased to exist and another has been created” (Starmans and Bloom 2018, 567). However, a recent comment by De Freitas and colleagues (2018) emphasizing the central role of moral attributes in personal identity suggests that such expressions may not be merely figurative but rather literal, such that there is indeed a meaningful sense in which pre-addiction and post-addiction Jim are numerically distinct.

We do not attempt to settle this issue here. However, we would like to offer that the two senses of identity may not be entirely conceptually separable. As Mott (2018) argues, part of the justification for statutory limitations on prosecuting certain crimes might be based in an intuitive recognition that after many years a person really does share less than the full identity of the transgressor (i.e., their past self), in some cases dropping below a threshold of qualitative similarity sufficient to sever the link of moral responsibility (see also Tobia, 2016). And in some cases, the sheer magnitude of dissimilarity between an agent before and after some transformative event may in fact break the identity relation in its stricter, numerical sense; philosophers disagree about such cases and the debate rages on (for an overview, see Glannon 1998; see also Shoemaker and Tobia, forthcoming).^11^

But perhaps these competing accounts can in fact be reconciled. One clue comes from work on “dual character” concepts, as described by Knobe and colleagues (Knobe, Prasada, and Newman 2013; Newman and Knobe 2018). A dual character concept is picked out by both (1) a set of concrete features sufficient for or typical of membership in the category and (2) a set of abstract values that that those features serve to realize. Take the concept *scientist* as an example. Insofar as it is a dual character concept, it could be right to say that although someone is technically a scientist—because she has a degree in science, conducts experiments, and publishes papers, thus exhibiting the relevant concrete features for category membership—she might nevertheless fail to be a true scientist if her work is not grounded in the abstract values that are essential to a scientific worldview (i.e., careful observation, critical thinking, updating beliefs in light of evidence, etc.).

Personal identity may be a similar sort of concept. Thus, it could be right to say of addicted Jim that there is a technical (i.e., numerical) sense in which he is the same person as pre-addicted Jim: After all, he inhabits the same body, has most of the same memories, and so on. But there may also be a deeper sense in which it is right to say that he isn’t the same person: Qualities that are central to what makes Jim the sort of person he really is deep down inside—in short, his true self—have in fact changed.^12^

To be clear, then, it is this latter sense of “not the same person” we take people to mean when they describe changed identity after addiction, and it is the sense we had in mind and attempted to measure in the experiments described in this article. Indeed, the very phrasing of one of the items in our main dependent measure presumes continuity of identity in the technical or numerical sense: When we ask participants the extent to which they agree with the sentence “Jim is now pretty different from what he used to be all about,” it is clear that “Jim” and “he” must in some sense be referring to the same person, or else the statement is incoherent.

## Practical implications

What are the practical implications of our findings? At this point, we can only speculate. However, there may be some insight for how treatment and recovery are ideally framed, in terms of personal identity. As the website for a treatment facility in Florida counsels, “You will likely see that if you’re in a relationship with a drug addict, they become a completely different person than the one you originally knew” upon recovering from the addiction (Recovery Village 2018). Indeed, our results support such a likelihood, as the character Jim was judged to be far closer to a “completely different” person than “exactly the same” person, not only when becoming addicted, as we had predicted and as we have emphasized throughout this article, but also when recovering from addiction.

In this context, it is easy to imagine feeling frightened by the prospect that the person you love—when that person is currently dealing with an addiction—might become a “completely different” person by getting treatment, even though the treatment is likely to make their life go better. Similarly, when addiction is part of an individual’s own “deep self-identification,” as Flanagan (2013) has discussed, the prospect of losing oneself through recovery might also be frightening, leading to a disinclination to seek treatment in the first place.^13^

But if that is the worry, our findings suggest it may be misplaced. First, becoming addicted to drugs consistently led to greater perceived identity change in our studies than recovering from addiction, suggesting that there is less to fear (in this regard) about the latter. And second, if recovery results in an improvement to a person’s moral character, although they may indeed superficially seem quite different to when they were dealing with addiction, on a deeper level they are likely to be seen as moving closer to their true self: to who they really are, deep down inside. That may be a more comforting thought.

If our results and this interpretation of them are on the right track, they might suggest that talking about treatment in terms of recovering—or perhaps discovering—one’s true self could be especially effective (for related work, see Schlegel and Hicks 2011; Schlegel et al. 2009, 2011). What we have in mind are messages like the following from The Canyon treatment center: “When you know that alcoholism and drug addiction has taken over your life, *get your identity back* by beginning drug treatment,” and “Drug treatment helps you *awaken your personality, character, and spirituality*” (The Canyon 2009, emphasis added). Similarly, another clinic notes that “for an individual to *reclaim their former self* after being affected by substance abuse, they need to be ready to commit to serious lifestyle changes, starting with quitting,” and “With a commitment to a healthy, more positive lifestyle, an addicted person can *surely find themselves again*” (Mountainside 2017, emphasis added). Indeed, we are encouraged by recent work in this vein suggesting that consideration of an out-group member’s true self can help to reduce intergroup bias (De Freitas and Cikara 2018). Insofar as people with addictions are considered part of an out-group, a focus on their good true self may suggest new strategies for mitigating the stigmas surrounding drug addiction—especially now that the “brain disease” strategy has, as we noted in the introduction, failed to stand up to empirical scrutiny.

## Limitations and future directions

Over a series of studies, we extended recent work in moral psychology and experimental philosophy to the more ecologically valid context of drug addiction. But these studies are only an initial step. For example, there are several variables of interest we chose not to manipulate—at least not systematically—for the sake of simplicity: Race, gender, sexual orientation, socioeconomic status, and so on, are all obvious examples, as they will undoubtedly interact in complex ways with judgments of identity change in the context of addiction. It may also be revealing to manipulate the source or kind of addiction: Does addiction to alcohol versus various kinds of drugs, or perhaps gambling, have different implications for perceptions of identity change? Another variable that should be manipulated in future studies is voluntariness: Presumably, addiction and drug use are more likely to be seen as morally bad when the agent appears capable of having done otherwise, as opposed to being forced by external pressures (including structural factors such as poverty). And finally, while we report incidental findings concerning the impact of a person’s intuitive model of addiction—i.e., medical versus moral—on judgements of identity change, distance from the true self, and responsibility in the supplementary materials, these issues require much more sustained and theoretically driven attention. We hope to contribute to such matters in future work.

## ACKNOWLEDGMENTS

We thank Kevin Tobia, Neil Levy, Hedy Kober, Brendan DeKenessey, Ryan Carlson, and the BMH Neuroscience and Society Group at Monash University (Adrian Carter, Ella Dilkes-Frayne, Andrew Dawson, Tony Barnett, Dan Myles, Cassandra Thomson, and Michelle James) for feedback on this work, and we especially thank Joshua Knobe for his considerable support and thoughtful input on the design, analysis, and interpretation of the studies reported in this article.

## AUTHOR CONTRIBUTIONS

BDE initially conceived of the studies with JACE, designed the materials, collected and analyzed the data, and wrote the initial draft of the report. JAS and JACE contributed substantially to the design and interpretation of the studies and to writing various portions of the article. JS contributed to the interpretation of the studies, framing of the discussion, and writing of various portions of the article. All authors contributed to multiple rounds of revision.

## FUNDING

Funding was provided by the Yale University Psychology Department. Additional funding was provided by a Wellcome Trust grant on healthcare and responsibility, #104848/Z/14/Z, to J.S.

## CONFLICTS OF INTEREST

None disclosed.

## ETHICAL APPROVAL

This study was considered exempt by the institutional review board at Yale University.

## Supplementary Material

Supplemental Material

## Footnotes

1 We note that MTurk samples tend to be more demographically diverse than traditional student samples, but they are not nationally representative (Buhrmester et al. 2011). Generalization is therefore not advised.

2 A theoretically uninteresting explanation for this concordance between conditions is that participants simply like to mark responses above the midpoint of a scale: Across all four items measuring *identity change*, greater change was always tied to the right-hand side of the scale, whereas less change was tied to the left. While this was done on purpose to be more intuitive to participants (i.e., conform to the left–right reading bias), it is possible that counterbalancing the left–right orientation of the scale would make a difference in future studies.

3 Note that one participant listed their age as 1987; this was recoded to 30 on the assumption that the person had entered their birth year by mistake.

4 For this replication attempt, 353 MTurkers were recruited (versus 290 in the original study), of whom 24 were excluded for failing the attention check. This left 329 participants (169 female, *M_age_* = 37.62, *SD* = 11.69). As before, the four identity change items formed a reliable scale (α = .90).

5 In this case, while there was no interaction effect between character and condition (*p* = .105), there was a main effect of character, *F*(4,624) = 2.60, *p* = .035, η_p_² = .016.

6 In contrast to the confirmatory results for identity change, there was a three-way interaction among character, condition, and drug status on true self judgments: *F*(4,1156) = 3.45, *p* = .008, η_p_² = .012; however, the very same pattern of significant two-way interactions between condition and drug status occurred for all five characters (all *p*s < .005), with only the effect sizes differing.

7 There was also an interaction between character and condition, *F*(4,533) = 2.49, *p* = .043, η_p_² = .018.

8 Another ambiguity with the previous studies concerned the amount of time Jim or the other characters had been characterized by certain attributes and behaviors (i.e., “for quite some time”) before the introduction or cessation of the drug, which made it difficult to know what baseline participants intuitively saw Jim or the others returning to or departing from in making their judgments. To remove this ambiguity, all vignettes now start with “For most of his life,” so that participants will have a more uniform basis for making their assessments of changed identity. Finally, in the bad drug conditions, rather than referring to the drug as “addictive” and simply assuming that participants will infer that it is an illegal, recreational substance, as in the earlier studies, we now explicitly describe the drug as such.

9 We are indebted to an anonymous referee for this helpful suggestion.

10 James Rachels (1987) has drawn a roughly similar distinction, between a biological life (numerical identity) and a biographical life (qualitative identity). The relevance here is that addiction can sometimes result in substantial changes to a person's biography, making them qualitatively different over time. And as Parfit (1984) argued, “what matters” for identity is not so much persistent association with the same body, but rather "psychological connectedness and/or psychological continuity, with the right kind of cause” (262). If we accept such a view, what defines ”me” has to do with my present mental states, and what is important for personal identity are the relationships of other mental states to those present mental states. In some cases, a person’s future self may be more like another person than it is like the present person, given sufficient change or discontinuity (e.g., as a result of addiction).

11 We note that much of the philosophical debate about personal identity has tended to focus on these kinds of practical concerns (e.g., desert, blame, etc.). We thank an anonymous referee for this helpful point.

12 This is, in principle, an empirical question. If participants are indeed operating with dual-character concepts, then they should, all else equal, be willing to endorse statements (in the context of the present study) such as “there is a sense in which the addicted man is still Jim, but there is also a sense in which he is not really Jim at all.” Thanks to an anonymous referee for this helpful suggestion.

13 An anonymous referee has pointed out that while this suggestion might be plausible, the results from our studies speak more directly to a third-personal perspective than a first-personal one (i.e., addicts themselves). Future research should explore the extent to which addicts think of themselves as “a different person” pre- and post-addiction.
