# Supplementary material for: Addiction, Identity, Morality
Source: AJOB Empir Bioeth. 2019 Apr 23;10(2):136–53. doi: 10.1080/23294515.2019.1590480 (PMC6506907; doi:10.1080/23294515.2019.1590480)
Supplement: Supplemental Material [file UABR_A_1590480_SM8940.docx]

**SUPPLEMENTARY MATERIALS**

**Study 1**

**Supplementary Method**

*Addiction Model*

Participants were shown the following statement: “There are two main ideas about what addiction is. One idea says that addiction is essentially a brain disease or a medical problem, having to do with certain changes that happen to the brain. The other idea says that addiction is more about weakness of will or a moral problem, having to do with making bad decisions. Which idea is closer to the one that you personally believe in?” Participants were then asked to select between: (1) Addiction is more like a ‘brain disease’ or a medical problem, and (2) Addiction is more like a weakness of will or a moral problem.”

**Supplementary Results**

To test for moderation, a 2 (condition: starting addiction, stopping addiction) X 2 (addiction model: medical, moral) ANOVA was conducted on identity change judgments. There was no main effect of addiction model (p = .393), nor was there an interaction between addiction model and condition (p = .256).

**Study 2**

**Vignettes**

*Becoming Addicted to a “Good” Drug (Starting Good)*. Jim never used to take medication, but now he needs to take one that has various side-effects on his feelings and behavior, so he's started taking it. About a year ago, some big changes happened in Jim’s life, and he needed to start taking the medication. Like most people who take this particular medication, Jim finds it very difficult to refrain from taking it, even when there are bad consequences. When he is prevented from taking his medication, he experiences very unpleasant feelings of withdrawal. He now spends a lot of his time thinking about, and seeking to refill the prescription for, his medication.

*Losing Addiction to a “Good” Drug (Stopping Good)*. Jim used to take a medication that had various side-effects on his feelings and behavior, but now he doesn't need to take it anymore, so he's stopped taking it. About a year ago, some big changes happened in Jim’s life, and he stopped needing to take the medication. Like most people who take this particular medication, Jim used to find it very difficult to refrain from taking it, even when there were bad consequences. When he was prevented from taking his medication, he experienced very unpleasant feelings of withdrawal. He used to spend a lot of his time thinking about, and seeking to refill the prescription for, the medication.

*Becoming Addicted to a “Bad” Drug (Starting Bad)*. [Identical to Starting Addiction in Study 1.]

*Losing Addiction to a “Bad” Drug (Stopping Bad)*. [Identical to Losing Addiction in Study 1.]

**Supplementary Method**

*Identity Change****.*** Participants were given, in random order, the same two questions and two statements described in Study 1. Cronbach’s α was .927. As in Study 1, a new variable, *Identity Change*, was created by mean-averaging the 4 items.

*Attention Check***.** The wording of the attention check was slightly different than in Study 1. In this study, to accommodate the new reference to medicine, it read: “… At the end of the story, was Jim still taking a substance or had he STOPPED taking it?” Participants were asked to pick between (1) “Jim was still taking a substance,” and (2) “Jim had STOPPED taking a substance.” Relevant participants were excluded as before.

**Study 3**

**Vignettes**

*Starting Bad*. For quite some time, Jim had been very motivated. Instead of spending his spare time lying around the house watching TV, he would always be working on his writing. He never missed shifts at his day job, making his boss really happy. His old friends saw him as truly reliable. At some point, Jim experimented with recreational drugs and found one that is pretty addictive. A little while ago he started regularly taking this drug. Now, he doesn't show up to work on time anymore, and sometimes he misses his shifts. He's started letting his friends down. And worst of all, he no longer spends his evenings at the local coffee shop writing poetry or perfecting his craft.

*Stopping Bad*. For quite some time, Jim had been very unmotivated. In fact, he used to spend almost all of his spare time lying around the house watching TV instead of working on his writing. Sometimes, he missed shifts at his day job, making his boss really angry. Even his old friends had come to see him as unreliable. At some point, Jim had experimented with recreational drugs and found one that is pretty addictive. A little while ago he stopped taking this drug. Now, he shows up to work on time and doesn't miss any shifts. He's stopped letting his friends down. And best of all, he spends his evenings at a local coffee shop writing poetry and perfecting his craft.

*Starting Good*. For quite some time, Jim had been very unmotivated. In fact, he used to spend almost all of his spare time lying around the house watching TV instead of working on his writing. Sometimes, he missed shifts at his day job, making his boss really angry. Even his old friends had come to see him as unreliable. Jim has a medical condition that affects his feelings and behavior, and there is a drug he can take to treat the underlying problem. A little while ago, he started regularly taking this drug. Now, he shows up to work on time and doesn't miss any shifts. He's stopped letting his friends down. And best of all, he spends his evenings at a local coffee shop writing poetry and perfecting his craft.

*Stopping Good*. For quite some time, Jim had been very motivated. Instead of spending his spare time lying around the house watching TV, he would always be working on his writing. He never missed shifts at his day job, making his boss really happy. His old friends saw him as truly reliable. Jim has a medical condition that affects his feelings and behavior, and there is a drug he was taking to treat the underlying problem. A little while ago he stopped taking this drug. Now, he doesn't show up to work on time anymore, and sometimes he misses his shifts. He's started letting his friends down. And worst of all, he no longer spends his evenings at the local coffee shop writing poetry or perfecting his craft.

**Supplementary Method**

Participants were asked a slightly different question compared to the previous study to account for the change in scenarios. They were asked to “Think about how Jim is right now, compared to how he was ‘for quite some time’ before the change with the drug. To what extent do you feel that Jim, as a person, has changed or stayed the same? On the next few pages you'll receive some questions and statements to try to get at your intuition.” Participants then answered the following questions, responded to an attention check, and filled out some basic demographic information. At the conclusion of the survey they were debriefed and thanked for their time.

*Identity Change****.*** Participants were given, in random order, the same two questions and two statements from the previous studies. As before, the items formed a reliable scale (α = .908).

*Attention Check***.** The same basic attention check as was used in Study 2 was used again here.

**Study 4**

**Vignettes**

See the complete materials at <https://osf.io/bm96x/>.

**Supplementary Method**

Participants were asked to “Think about how the character from the story is right now, compared to how they were ‘for quite some time’ before the change with the drug. To what extent do you feel that the character, as a person, has changed or stayed the same? On the next few pages you'll receive some questions and statements to try to get at your intuition.” Participants then answered the following questions, responded to an attention check, and filled out some basic demographic information. At the conclusion of the survey they were debriefed and thanked for their time.

*Identity Change****.*** Participants were given, in random order, the same two questions and two statements from the previous studies. As before, the items formed a reliable scale (α = .92).

*True Self*. Participants were asked, “Would you say that now, compared to at the beginning of the story, the character is closer to, or farther away from, being their TRUE SELF, that is, who they most essentially are, deep down?” They were then shown a scale ranging from 0 to 100, with 0 labeled, “Much closer to” and 100 labeled “Much farther away,” on which they recorded their answer.

*Responsibility****.*** Participants were asked, “To what extent was the character responsible for their behavior while they were regularly taking the drug?​” they were then shown a scale ranging from 0 to 100 with 0 labeled, “Not at all responsible” and 100 labeled “Completely responsible,” on which they recorded their answer.

*Addiction Model****.*** Participants were asked to indicate whether they personally believe more in a “moral” or “medical” model of addiction, using the same question as in Study 1.

*Attention Check***.** Participants were told: “This is the last question, just to check if you remember the story about the character. At the **end** of the story, was the character still taking a drug, or had they STOPPED taking it?” They then were then asked to pick between, “They were taking a drug” and “They had STOPPED taking a drug.” Participants who chose the incorrect answer based on their condition were excluded from all further analyses.

**Supplementary Results**

*Responsibility*

A 2 (condition) by 2 (drug status) by 5 (character) ANOVA was conducted on responsibility judgments. As with ientity change and true self, there was a significant main effect of drug status on responsibility judgments, F(1, 1158) = 11.02, p = .001, ηp² = .01, with greater judged responsibility for one’s behavior while under the influence of bad drugs (M = 77.49, SD = 23.95) than good drugs (M = 72.86, SD = 24.01). There was no main effect of condition (p = .907) and there were no significant interactions (all ps > .316).

There was also a main effect of character, *F*(4,1158) = 3.48, *p* = .008, η_p_² = .01. On a separate note, please recall that, for responsibility judgments, participants were *not* asked to judge the character’s responsibility for their actions at Time 2 (i.e., having either started or stopped taking the good or bad drug) compared to Time 1 (i.e., the baseline). Rather, they were asked to judge the character’s responsibility for their actions *while they were under the influence* of the relevant drug, which was at Time 1 for the Stopping vignettes, and Time 2 for the Starting vignettes.

*Addiction model*

As an additional exploratory analysis, as pre-registered, we sought to determine whether the model of addiction one endorses moderates the relationship between condition and our main outcome variable, identity change. Accordingly, we conducted a 2 (condition) by 2 (drug status) by 2 (addiction model) by 5 (character) ANOVA on identity change judgments. There was no main effect of addiction model (p =.708), nor did it interact with any of the other variables in predicting judgments of identity change (all ps > .073).

Next, although we had not pre-registered the following analyses, we decided to see whether preferred addiction model moderated the relationship between condition and responsibility judgments, given the close conceptual relationship between these variables we noted above. We therefore conducted a 2 (condition) by 2 (drug status) by 2 (addiction model) by 5 (character) ANOVA on responsibility judgments. In this case, there was a main effect of addiction model, F(1,1138) = 7.75, p = .005, ηp² = .007, such that participants who favored the moral model tended to judge characters as being more responsible for their behavior while under the influence of any drug (M = 78.37, SD = 24.73) compared to participants who favored the medical model (M = 73.73, SD = 23.57). However, the effect size for this finding is extremely small (ηp² = .007). There were also no interactions between addiction model and any of the other variables (all ps > .075).

For the sake of completeness, we conducted the same 2 X 2 X 2 X 5 ANOVA on true self judgments. There was no main effect of addiction model (p = .708), nor did addiction model interact with any of the other variables (all ps > .072).

**Supplementary Discussion**

Given such a high-powered study, the fact that participants’ favored addiction model (medical versus moral) failed to moderate the relationship between condition and any of the dependent variables (identity change, true self, responsibility), is perhaps surprising, as is the smallness of the main effect of addiction model on judgments of responsibility. As alluded to in the Introduction much ado has been made (by some) about the need to promote a medical or brain-disease model of addiction, lest people too harshly judge persons with addiction in virtue of assigning them personal responsibility for their plight (Leshner 1997). But here, the model of addiction one personally endorses did *not* seem to make a difference to identity judgements or true self judgements, and it made a very tiny difference to judgements of responsibility. Much more important for shaping participant intuitions about responsibility was the goodness or badness of the direction of change of moral character.

**Study 5**

**Vignettes**

*Bad Drug 🡪 Bad Person*. For most of his life, Jim was very motivated, reliable, and kind to others. In fact, he would never spend his spare time just lying around the house watching TV, but would instead be very selfless and often help people in need. He rarely missed shifts at his job, making his boss really happy. Even strangers saw him as truly trustworthy and kind-spirited. At some point, Jim experimented with illegal, recreational drugs. A little while ago he started regularly taking one of these drugs which is unfortunately very addictive. It makes Jim feel really pleasurable when he is "on" it, and it causes painful withdrawal symptoms when he doesn't take it. He thinks about using the drug all the time. As an additional side-effect of the drug, Jim has become a lot less motivated, reliable, and kind. He now doesn't show up to work on time and often misses his shifts. He's started letting his friends down and is much less helpful to others. He's even stopped volunteering at the local soup kitchen where he used to spend his weekends. Everyone agrees that Jim is a lot meaner and less moral than he used to be before he started using the drug.

*Bad Drug 🡪 Good Person*. For most of his life, Jim was very unmotivated, unreliable, and unkind to others. In fact, he used to spend almost all of his spare time lying around the house watching TV, while being very selfish and rarely helping people in need. Sometimes, he missed shifts at his job, making his boss really angry. Even his own relatives saw him as untrustworthy and mean-spirited. At some point, Jim experimented with illegal, recreational drugs. A little while ago he started regularly taking one of these drugs which is unfortunately very addictive. It makes Jim feel really pleasurable when he is "on" it, and it causes painful withdrawal symptoms when he doesn't take it. He thinks about using the drug all the time. As a weird side-effect, however, Jim has become a lot more motivated, reliable, and kinder. He now shows up to work on time and doesn't miss any shifts. He's stopped letting his friends down and is much more helpful to others. He's even started volunteering at a local soup kitchen over the weekends. Everyone agrees that Jim is a lot nicer and more moral than he used to be before he started using the drug.

*Good Drug 🡪 Good Person*. For most of his life, Jim was very unmotivated, unreliable, and unkind to others. In fact, he used to spend almost all of his spare time lying around the house watching TV, while being very selfish and rarely helping people in need. Sometimes, he missed shifts at his job, making his boss really angry. Even his own relatives saw him as truly untrustworthy and mean-spirited. At some point, Jim had to go on medication for an unrelated issue where he would occasionally get rashes on his skin. A little while ago he started regularly taking the medication for this skin problem which is unfortunately very addictive. It makes Jim feel really pleasurable when he is "on" it, and it causes painful withdrawal symptoms when he doesn't take it. He thinks about taking the medication all the time. As a weird side-effect of the medication, however, Jim has become a lot more motivated, reliable, and kinder. He now shows up to work on time and doesn't miss any shifts. He's stopped letting his friends down and is much more helpful to others. He's even started volunteering at a local soup kitchen over the weekends. Everyone agrees that Jim is a lot nicer and more moral than he used to be before he started using the medication.

*Good Drug 🡪 Bad Person*. For most of his life, Jim was very motivated, reliable, and kind to others. In fact, he would never spend his spare time just lying around the house watching TV, but would instead be very selfless and often help people in need. He rarely missed shifts at his job, making his boss really happy. Even strangers saw him as truly trustworthy and kind-spirited. At some point, Jim had to go on medication for an unrelated issue where he would occasionally get rashes on his skin. A little while ago he started regularly taking the medication for this skin problem which is unfortunately very addictive. It makes Jim feel really pleasurable when he is "on" it, and it causes painful withdrawal symptoms when he doesn't take it. He thinks about taking the medication all the time. As an additional side-effect, Jim has become a lot less motivated, reliable, and kind. He now doesn't show up to work on time and often misses his shifts. He's started letting his friends down and is much less helpful to others. He's even stopped volunteering at the local soup kitchen where he used to spend his weekends. Everyone agrees that Jim is a lot meaner and less moral than he used to be before he started using the medication.

**Supplementary Method**

Participants were then asked to “Think about how Jim is right now, compared to how he was ‘for most of his life’ before he started taking the drug. To what extent do you feel that Jim, as a person, has changed or stayed the same? On the next few pages you'll receive some questions and statements to try to get at your intuition.” Participants then answered the following questions, responded to an attention check, and filled out some basic demographic information. At the conclusion of the survey they were debriefed and thanked for their time.

*Identity Change****.*** Participants were given, in random order, the same two questions and two statements from the previous studies. As before, the items formed a reliable scale (α = .924).

*True Self*. As in the previous study, participants were asked, “Would you say that now, compared to at the beginning of the story, the character is closer to, or farther away from, being their TRUE SELF, that is, who they most essentially are, deep down?” They were then shown a scale ranging from 0 to 100, with 0 labeled, “Much closer to” and 100 labeled “Much farther away,” on which they recorded their answer.

*Addiction Model****.*** Participants were asked to indicate whether they personally believe more in a “moral” or “medical” model of addiction, using the same question as in Study 1.

*Attention Check***.** The same basic attention check as was used in previous studies was used again here.

**Supplementary Results**

*Addiction model*

As an exploratory analysis, to see whether participants’ preferred addiction model moderated the relationship between condition and the dependent measures, we ran two separate 2 (drug status) by 2 (person status) by 2 (addiction model) ANOVAs, one on identity change judgments and one on true self judgments.

Starting with identity change judgments, while there was no main effect of addiction model (p = .743), there was a three-way interaction among drug status, person status, and addiction model, F(1,567) = 5.54, p = .019, ηp² = .01. To break this down, we ran two separate 2 (drug status) by 2 (person status) ANOVAs, looking first at participants who endorsed the medical model (N = 394, or 68.4% of the sample), and second at those who endorsed the moral model (N = 181, or 31.4% of the sample).

Among the former, curiously, there was no main effect of drug status, nor a main effect of person status, nor an interaction between the two (all ps > .159). By contrast, among participants who endorsed the moral model, while there was no main effect of drug status (p = .593), there was a main effect of person status, F(1,177) = 6.47, p = .012, ηp² = .04, such that Jim was judged has having undergone less identity change when he became a good person (M = 69.52, SD = 23.59) compared to when he became a bad person (M = 78.70, SD = 23.02).

There was also a significant interaction between drug status and person status: F(1,177) = 8.70, p = .004, ηp² = .05. Looking just within the good drug conditions, a t-test showed that participants judged Jim to have undergone less identity change when he became a good person (M = 73.89, SD = 18.99) compared to a bad person (M = 76.04, SD = 18.78), though this difference was not statistically significant: t(201) = -.807, p = .421, d = .01. Within the bad drug conditions, participants similarly judged Jim to have undergone less identity change when he became a good person (M = 69.99, SD = 24.64) compared to a bad person (M = 73.93, SD = 20.36), though this, too, was not statistically significant: t(189) = -1.17, p = .242, d = .17.

Moving now to true self judgments, while there was no main effect of addiction model (p = .976), nor an interaction between addiction model and drug status (p = .663), or among addiction model, drug status, and person status, (p = .137), there was a two-way interaction between addiction model and person status, F(1,576) = 4.66, p = .031, ηp² = .01. However, regardless of addiction model, Jim was judged as being much closer to his true self when he became a good person compared to a bad person, suggesting that the interaction was driven by a difference in means in the same direction rather than a different overall pattern of results.

Looking just at participants who endorsed the medical model, there was a main effect of goodness/badness of person on True Self judgments, such that Jim was judged as being much closer to his True Self when he became a good person (*M* = 61.62, *SD* = 27.77) compared to when he became a bad person (*M* = 83.22, *SD* = 18.58), *F*(1,390) = 76.15, *p* < .001, η_p_² = .16. Similarly, looking just at participants who endorsed the moral model, there was a main effect of goodness/badness of person on True Self judgments, such that Jim was judged as being much closer to his True Self when he became a good person (*M* = 66.46, *SD* = 25.99), compared to when he became a bad person (*M* = 78.49, *SD* = 24.21), *F*(1,177) =9.91, *p* = .002, η_p_² = .05.

**Study 6**

**Vignettes**

*Endorses Bad.* Jim is 27 years old. He graduated from Briarcrest High School in a town called Bloomington when he was 17. Since then, he’s attended community college, traveled some, worked different jobs, and learned how to play the guitar.

If there is one big thing that Jim doesn’t like about himself, it’s that he’s not very tough. When he watches movies, he idealizes the ‘bad boy’ characters — the ones who put themselves first, act on impulse, and sometimes even get into trouble. He has thought about it a lot, and when it comes right down to it, Jim wishes he were just like those characters: hard as nails and a little bit wild.

But as it happens, Jim’s basic instincts and desires are unfailingly moderate and wholesome. He’s a pretty empathic and reliable friend. He shows up to work on time. He spontaneously helps other people out. He just doesn’t crave bad, reckless things, no matter how much he would like to. So from his perspective, he’s stuck being boring, “good old Jim.”

Now, Jim has a long-standing medical condition where he occasionally gets irritating rashes on his skin. A little while ago he started taking a drug to address this skin problem. This drug works well and Jim no longer gets the rashes.

Yet as a side-effect of the drug, Jim has become much more impulsive. He’s started shoplifting just for the fun of it. He cancels on his friends at the last minute. He’s often late to work, and sometimes misses his shifts. He picks fights with people at the slightest provocation. Everyone agrees that Jim is a lot less kind and helpful than he used to be before he started on the drug.

*Rejects Good.* Jim is 27 years old. He graduated from Briarcrest High School in a town called Bloomington when he was 17. Since then, he’s attended community college, traveled some, worked different jobs, and learned how to play the guitar.

If there is one big thing that Jim likes about himself, it’s that he’s very tough. When he watches movies, he idealizes the ‘bad boy’ characters — the ones who put themselves first, act on impulse, and sometimes even get into trouble. He has thought about it a lot, and when it comes right down to it, Jim is glad he is just like those characters: hard as nails and a little bit wild.

As it happens, Jim’s basic instincts and desires are often intense and unwholesome. He’s not a particularly empathic or reliable friend. He stays out all night and shows up late to work. He shoplifts things just for the fun of it. He picks fights with people at the slightest provocation. He just craves bad, reckless things, no matter how much others wish he didn’t. From his perspective, at least he’s not some boring, “good old Jim.”

Now, Jim has a long-standing medical condition where he occasionally gets irritating rashes on his skin. A little while ago he started taking a drug to address this skin problem. This drug works well and Jim no longer gets the rashes.

Yet as a side-effect of the drug, Jim has become much less impulsive. He’s stopped shoplifting and returned all the stolen goods. He now shows up to work on time. He's there for his friends more when they need him, and he doesn’t pick fights. Everyone agrees that Jim is a lot more kind and helpful than he used to be before he started on the drug.

*Rejects Bad.* Jim is 27 years old. He graduated from Briarcrest High School in a town called Bloomington when he was 17. Since then, he’s attended community college, traveled some, worked different jobs, and learned how to play the guitar.

If there is one big thing that Jim likes about himself, it’s that he’s not very tough. When he watches movies, he never idealizes the ‘bad boy’ characters — the ones who put themselves first, act on impulse, and sometimes even get into trouble. He has thought about it a lot, and when it comes right down to it, Jim is glad he is not like those characters: hard as nails and little bit wild.

As it happens, Jim’s basic instincts and desires are unfailingly moderate and wholesome. He’s a pretty empathic and reliable friend. He shows up to work on time. He spontaneously helps other people out. He just doesn’t crave bad, reckless things, like some people do. From his perspective, he’s perfectly happy being “good old Jim.”

Now, Jim has a long-standing medical condition where he occasionally gets irritating rashes on his skin. A little while ago he started taking a drug to address this skin problem. This drug works well and Jim no longer gets the rashes.

Yet as a side-effect of the drug, Jim has become much more impulsive. He’s started shoplifting just for the fun of it. He cancels on his friends at the last minute. He’s often late to work, and sometimes misses his shifts. He picks fights with people at the slightest provocation. Everyone agrees that Jim is a lot less kind and helpful than he used to be before he started on the drug.

*Endorses Good*. Jim is 27 years old. He graduated from Briarcrest High School in a town called Bloomington when Q19 he was 17. Since then, he’s attended community college, traveled some, worked different jobs, and learned how to play the guitar.

If there is one big thing that Jim doesn’t like about himself, it’s that he’s very tough. When he watches movies, he never idealizes the ‘bad boy’ characters — the ones who put themselves first, act on impulse, and sometimes even get into trouble. He has thought about it a lot, and when it comes right down to it, Jim wishes he were not like those characters: hard as nails and a little bit wild.

But as it happens, Jim’s basic instincts and desires are often intense and unwholesome. He’s not a particularly empathic or reliable friend. He stays out all night and shows up late to work. He shoplifts things just for the fun of it. He picks fights with people at the slightest provocation. He just craves bad, reckless things, no matter how much he wishes he didn’t. From his perspective, he would much rather be a regular, “good old Jim.”

Now, Jim has a long-standing medical condition where he occasionally gets irritating rashes on his skin. A little while ago he started taking a drug to address this skin problem. This drug works well and Jim no longer gets the rashes.

Yet as a side-effect of the drug, Jim has become much less impulsive. He’s stopped shoplifting and returned all the stolen goods. He now shows up to work on time. He's there for his friends more when they need him, and he doesn’t pick fights. Everyone agrees that Jim is a lot more kind and helpful than he used to be before he started on the drug.
